# Outcomes and costs of Ranibizumab and Aflibercept treatment in a health-service research context

**DOI:** 10.1186/s12886-018-0731-4

**Published:** 2018-02-27

**Authors:** Martin K. Schmid, Oliver Reich, Eva Blozik, Livia Faes, Nicolas S. Bodmer, Silvan Locher, Michael A. Thiel, Roland Rapold, Maximilian Kuhn, Lucas M. Bachmann

**Affiliations:** 1 University of Zurich, Zurich, Switzerland; 2 Eye Clinic, Cantonal Hospital of Lucerne, Lucerne, Switzerland; 3 Department of Health Sciences, Helsana Group, Zurich, Switzerland; 4 Medignition Inc. Research Consultants, Verena Conzett-Strasse 9, P.O. 9628, 8036 Zurich, CH Switzerland; 5 CSS Group, Luzern, Switzerland

**Keywords:** Claims data, Health insurance, Cost analysis, Macular degeneration, Aflibercept, Ranibizumab

## Abstract

**Background:**

To compare anti-VEGF treatments for macular disease in terms of costs and clinical outcomes.

**Methods:**

We identified patients suffering from macular disease and treated either with aflibercept, ranibizumab or both at the largest public eye clinic in Switzerland between January 1st and December 31st 2016 who were insured in one of the two participating health insurance companies. Clinical data were extracted from the electronic health record system. The health insurers provided the health claim costs for the ophthalmologic care and the total health care costs of each patient in the observation period. Using multivariate regression models, we assessed the monthly ophthalmologic and the monthly total costs of patients with no history of switching (ranibizumab vs. aflibercept), patients with a history of switching from ranibizumab to aflibercept, patients switching during the observation period and a miscellaneous group. We examined baseline differences in age, proportion of males, visual acuity (letters), central retinal thickness (CRT) and treatment history before entering the study. We investigated treatment intensity and compared the changes in letters and CRT.

**Results:**

The analysis involved 488 eyes (361 patients), 182 on ranibizumab treatment, and 63 on aflibercept treatment, 160 eyes with a history of switching from ranibizumab to aflibercept, and 45 switchers during follow-up and 38 eyes of the miscellaneous group. Compared to ranibizumab, monthly costs of ophthalmologic treatment were slightly higher for aflibercept treatment + 175.0 CHF (95%CI: 1.5 CHF to 348.3 CHF; *p* = 0.048) as were the total monthly costs + 581.0 CHF (95%CI: 159.5 CHF to 1002.4 CHF; *p* = 0.007). Compared to ranibizumab, the monthly treatment intensity with aflibercept was similar (+ 0.057 injections/month (95%CI -0.023 to 0.137; *p* = 0.162), corresponding to a projected annual number of 5.4 injections for ranibizumab vs. 6.1 injections for aflibercept. During follow-up, visus dropped by 0.7 letters with ranibizumab and increased by 0.6 letters with aflibercept (*p* = 0.243). CRT dropped by − 14.9 μm with ranibizumab and by − 19.5 μm with aflibercept (*p* = 0.708). The monthly costs of all other groups examined were higher.

**Conclusion:**

These real-life data show that aflibercept treatment is equally expensive, and clinical outcomes between the two drugs are similar.

## Background

Two years ago, we published a study comparing the reimbursed treatment costs and clinical outcomes of ranibizumab and aflibercept in the treatment of macular conditions in Switzerland, when adjusting for patients’ characteristics and clinical status [1]. We found that the two anti-VEGF medications do not differ in clinical outcomes, injection frequency and costs. Differences in costs could be explained by the underlying clinical condition. Also, patients’ characteristics and duration of medication were associated with the variability in cost. The study also showed that aflibercept and ranibizumab were used in a similar fashion in Switzerland when applying the same treatment scheme, which was unexpected in view of the fact that aflibercept was assumed to require less injections. Consequently the total health care expenditures for both these anti-VEGF agents [2] were comparable.

A major drawback of our study was the low number of patients receiving aflibercept overall and de novo. Consequently, the comparison between the two drugs lacked precision and robustness. To verify our initial findings we decided to repeat and expand the analysis by adding the health claims data of a second, large health insurance company. By interconnecting health care data with clinical data, we expected to find a solid depiction of the actual status quo in the real-life treatment of patients. In this study, we compared the reimbursed costs and clinical follow-up of ranibizumab and aflibercept treatment considering differences in patients’ characteristics and clinical status in the analysis.

## Methods

This study received Ethics approval from the Ethics Commission for North-East and Central Switzerland (EKNZ 2014–110 Amendment) and adhered to the Declaration of Helsinki and the principles of good clinical practice [3].

### Clinical visits

All patients followed an optical coherence tomography (OCT) guided pro re nata [4] treatment pattern. Accordingly, patients were seen on a monthly basis at the hospital’s retinal service. A fundoscopy, visual acuity (ETDRS) and OCT scanning (Spectralis, Heidelberg Engineering GmbH, 69,121 Heidelberg, Germany) was performed at each visit. The final decision for an injection was based on intra or subretinal fluid found in the OCT and an injection was made on the same day. Patients missing a follow-up visit were approached by the clinic to settle a new visit date. The clinical data were entered into the clinic’s electronic health record system (EHRS).

### Patient identification and matching

Patients with a Helsana or a CSS health insurance in the year 2016 who received either ranibizumab or aflibercept at the eye clinic were considered. From the electronic health record system, we obtained all available clinical data of these patients, also the data of previous years. On the other hand, Helsana and CSS provided the corresponding health care claims data of these patients for the same observation period. The databases were matched into one analysis file. We checked the quality of matching by comparing the health claims of the hospital’s system and the health insurer’s database. If the entries were inconsistent, we matched the clinical recordings of a specific visit to the entry date of the corresponding health claim, if the discrepancy between the two dates was less than 30 days. The data management adhered to the current data protection protocols and the requirements of the Ethics committee. Patient information was anonymized and de-identified prior to analysis.

### Clinical data

The target condition, gender and age, the visual acuity and retinal thickness (CRT) at study entry, the number of IVI per treatment, the visual outcome and the central retinal thickness (CRT) at the last visit of follow-up was well as vital status were secured. The medical history prior to the observation period in terms of treatment duration, number of injections and treatment changes were also extracted. The data for these parameters were complete. We used the data of both eyes if a patient had a binocular condition. The minimum follow-up period was 1 month.

### Outcomes

The outcome parameter total costs comprised the total of health care claims of 2016 in the numerator and number of months of follow-up in the denominator. This outcome was chosen to study global treatment effects within the various clinical groups. Costs for ophthalmologic treatment included all health care claims of the eye clinic that could be directly attributed to the anti-VEGF management in the numerator and duration of follow-up (months) in the denominator.

### Statistical analysis

Continuous variates were summarised with means, standard deviations and ranges and dichotomous variates were summarized with percentages. In univariate analyses, the association between clinical parameters and costs were examined. Between groups comparisons of continuous variates were made using the parametric t-test. Dichotomous variates were compared using the non-parametric chi-square test. Cost per month was computed by dividing the sum of costs (total or for eye treatment) with the number of month in the follow-up. These costs parameters were used throughout the analyses.

Statistical modelling was made on the level of eyes rather than patients. Normality of the error distribution was confirmed visually and statistically using the Anderson-Darling test. In separate multivariate linear regression models we compared global costs/month and ophthalmologic costs/month (dependent variables) between different patient-groups using four indicator variables for five therapies (only ranibizumab, only aflibercept, switchers from ranibizumab to aflibercept prior to the observation period, switchers to aflibercept during the observation period and a miscellaneous group) (independent variates). We adjusted for differences in the duration of treatment prior to study entry, patients’ age and female gender as well as for visual acuity at study entry and the CRT. We repeated these analyses for the subgroup of patients with diabetic macular edema. Statistical analyses were performed using the Stata 14.2 statistics software package. (StataCorp. 2015. Stata Statistical Software: Release 14. College Station, TX: StataCorp LP.)

## Results

### Patients’ characteristics

The analysis involved 488 eyes (361 patients), 182 eyes on ranibizumab treatment, and 63 eyes on aflibercept treatment, 160 eyes with a history of switching from ranibizumab to aflibercept prior to study entry, 45 switchers during follow-up and 38 eyes of the miscellaneous group including 13 patients with a double switch (ranibizumab, aflibercept, ranibizumab) before entering the study. Median follow-up period was 11 months (Interquartile range 11 to 12 months), mean age was 78.2 years (standard deviation (SD) 9.5), and 61.8% of all patients were female. In the ranibizumab group, 21 eyes (11.5%), and in the aflibercept group 8 eyes (12.7%) had a diabetic macular edema (DME) (*p* = 0.806). Among switchers prior to study entry (15.6%) and during the observation period (17.8%) the proportion of DME was similar. The miscellaneous group had the highest proportion of DME (18.4%).

At study entry, mean visus in letters was 65.3 letters (SD 24.0) and CRT was 318.5 (SD 106.6). Compared to ranibizumab, patients receiving aflibercept were significantly younger (75.1 years vs. 80.0, *p* < 0.001), and were more often male (46.0% vs. 34.6%, *p* = 0.107). They had a slightly better visus (69.4 letters vs. 64.7 letters, *p* = 0.159) and had a higher CRT (361.2 μm vs. 314.3 μm, *p* = 0.006) at study entry. Anti-VEGF treatment was initiated in 60 eyes during the observation period. Of them, 38 eyes (18.4%) started with ranibizumab and 22 eyes (9.3%) started with aflibercept (*p* = 0.005). the salient patient characteristics is shown in Table 1.

**Table 1:** Shows the distribution of salient clinical characteristics of the different treatment groups at study entry

Therapy	# Eyes (patients)	Age (sd^a^)	Male (%)	Visus (sd^a^)	CRT (sd^a^)	DME (%)	CRVO (%)	Mean # IVI before	Treatment duration prior study [months]
Only ranibizumab	182 (144)	80.0 (8.9)	63 (34.6)	64.7 (23.7)	314.3 (100.3)	21 (11.5)	7 (3.8)	8.7	19.5
Only aflibercept	63 (52)	75.1 (9.4)	29 (46.0)	69.4 (18.8)	361.2 (152.3)	8 (12.7)	6 (9.5)	4.7	7.9
*p*-values		< 0.001	0.107	0.159	0.006	0.806	0.083	0.002	< 0.001
Switching from ranibizumab to aflibercept prior study	160 (128)	77.4 (10.2)	66 (41.3)	65.8 (25.4)	304.0 (86.7)	25 (15.6)	7 (4.4)	27.4	51.3
Switching from ranibizumab to aflibercept during study	45 (36)	76.8 (8.7)	19 (42.2)	66.7 (23.8)	323.2 (93.9)	8 (17.8)	6 (13.3)	9.9	19.1
Miscellaneous^b^	38 (34)	78.9 (8.5)	10 (26.3)	57.2 (26.7)	302.7 (64.3)	7 (18.4)	2 (5.3)	31.8	63.5

^a^sd = standard deviation

^b^including eyes with various treatment regimens. The largest group consisted of 13 eyes with a double switch from ranibizumab to aflibercept and back to ranibizumab prior to study entry

### Assessment of costs

Compared to ranibizumab (*n* = 182 eyes), monthly costs of ophthalmologic treatment were slightly higher for aflibercept (*n* = 63 eyes) treatment + 175.0 CHF (95%CI: 1.5 CHF to 348.3 CHF; *p* = 0.048) as were the total monthly costs + 581.0 CHF (95%CI: 159.5 CHF to 1002.4 CHF; *p* = 0.007). When excluding patients with DME from this analysis, the monthly costs of ophthalmologic treatment were almost identical (+ 54.6 (95% CI: -118.1 CHF to 227.14 CHF; *p* = 0.534), while the total monthly cost remained slightly higher in the aflibercept (*n* = 55 eyes) group (+ 574.2 CHF (95% CI: 164.6 to 983.8; *p* = 0.006) compared to the ranibizumab group (*n* = 161 eyes). Irrespective of treatment, patients with DME had slightly, albeit not significantly higher, mean monthly costs for ophthalmologic (1284 CHF vs. 1224 CHF; *p* = 0.483) and mean total monthly costs (2412 CHF vs. 2321 CHF; *p* = 0.642). There was no interaction between treatment (ranibizumab or aflibercept) and presence of DME in respect to mean monthly ophthalmologic (*p* = 0.576) and mean monthly total costs (*p* = 0.386).

### Frequency of treatment and clinical follow-up

Compared to ranibizumab, the monthly treatment intensity with aflibercept was similar (+ 0.057 injections/month (95%CI -0.023 to 0.137; *p* = 0.162), corresponding to a projected annual number of 5.4 injections for ranibizumab vs. 6.1 injections for aflibercept. When excluding patients with DME the monthly injection frequency was almost identical (+ 0.02 injections/month (95% CI: -0.06 0.10; *p* = 0.619).

During follow-up, visus dropped by 0.7 letters with ranibizumab and increased by 0.6 letters with aflibercept (*p* = 0.243). CRT dropped by − 14.9 μm with ranibizumab and by − 19.5 μm with aflibercept (*p* = 0.708).

### Patients switching from ranibizumab to aflibercept

Compared to ranibizumab, ophthalmologic and total monthly costs of those switching from ranibizumab to aflibercept prior to study entry were significantly higher (+ 362.8 CHF (95CI: 204.9 CHF to 520.63 CHF; *p* < 0.001) and 443.2 CHF (95%CI; 70.8 CHF to 815.6 CHF; *p* = 0.020). Similarly, the ophthalmologic costs of those switching from ranibizumab to aflibercept during the observation period were + 269.0 CHF (95%CI: 61.7 CHF to 476.2 CHF; *p* = 0.011), but total monthly costs were slightly albeit not significantly higher (+ 378.8 CHF (95%CI: -110.1 CHF to 867.7; *p* = 0.129). The higher ophthalmologic costs were due to the higher treatment intensity compared to ranibizumab (+ 0.14 injections/month (95% CI: 0.06 to 0.22; *p* < 0.001) and + 0.16 injections/months (95%CI: 0.08 to 0.24; *p* < 0.001), corresponding to a mean of 6.5 injections and 7.4 injections per year, respectively.

### Miscellaneous group

In terms of treatment history, this was a heterogeneous group. The largest subgroup consisted of 13 eyes with a double switch (ranibizumab – aflibercept – ranibizumab) before entering the study. These patients were older (mean age 81.9 years (Range 75 to 94 years), had a lower visus (mean 57.4 letters (range 14 to 87 letters), had a mean CRT of 291.8 μm (range 214 to 432 μm) had received anti VEGF treatment for over 5 years (mean treatment duration in months 63.2 (range 36 to 93) with a mean of 39.4 injections (21 to 72). The mean monthly and total costs were 1308.9 CHF (range 515.5 to 2146.7 CHF) and 2280.7 CHF (range 661.5 to 3930.6 CHF) respectively. A summary of number of IVI and (unadjusted) monthly costs is shown in Table 2.

**Table 2:** Shows the distribution of number of IVI, mean monthly ophthalmologic and total cost of the different treatment groups. Note that these are unadjusted values

Therapy	# Eyes (patients)	Mean # IVI (sd^a^)	Ophthalmologic costs per months [CHF] (sd^a^)	Total costs per months [CHF] (sd^a^)
Only ranibizumab	182 (144)	4.3 (2.3)	1063.9 (549.3)	2060.5 (1274.4)
Only aflibercept	63 (52)	5.1 (2.5)	1238.8 (735.6)	2641.5 (1912.3)
Switching from ranibizumab to aflibercept prior study	160 (128)	5.9 (2.7)	1336.1 (742.2)	2486.8 (1702.0)
Switching from ranibizumab to aflibercept during study	45 (36)	6.9 (2.4)	1337.1 (443.1)	2408.5 (1133.6)
Miscellaneous^b^	38 (34)	6.6 (3.0)	1470.8 (770.5)	2404.5 (1141.9)

^a^sd = standard deviation

^b^including eyes with various treatment regimens. The largest group consisted of 13 eyes with a double switch from ranibizumab to aflibercept and back to ranibizumab prior to study entry

## Discussion

### Main findings

The results of this study show that aflibercept is equally expensive as ranibizumab in a pro re nata treatment scheme, while clinical outcomes between the two drugs are similar, when correcting for possible confounding due to differences in baseline characteristics. The higher total monthly cost in the aflibercept group may be due to the slightly higher number of patients with DME in this group.

### Results in context of the existing literature

Both the efficacy [5–7] and cost-effectiveness [8, 9] of ranibizumab and aflibercept has been thoroughly studied. A paper by Johnston and co-workers assessed first-line anti-VEGF management patterns in AMD using claims data [10]. They found no differences between the two drugs for number of injections and healthcare costs. Very recently, two studies assessed the efficacy of ranibizumab and or aflibercept in the clinical routine [11, 12]. One study found a similar effect of aflibercept as reported in the pivotal clinical studies [11] and Rasmussen, via retrospective chart review, found a 15% reduction in treatment frequency among patients receiving aflibercept rather than ranibizumab [12].

The findings presented here mostly corroborate those of our previous study [1]. By including a larger number of patients receiving aflibercept de novo, the study also overcomes a relevant shortcoming our previous report. While the pervious study showed equivalence between the two drugs, this update showed a small, albeit statistically significant higher costs at a similar clinical outcome. Again, patients receiving aflibercept were younger and had a slightly better vision at study entry. Why selection occurs, remains unclear. The use of either ranibizumab or aflibercept was fully at the discretion of the treating physician. Also, total costs for patients with DME was not as high as in the previous study, which may be due to the mean shorter observation period of 11 vs. 33 months. Patients with a history of switching or switching during the observation period were clinically different to those staying on either ranibizumab or aflibercept. Also, treatment costs were higher in the switching groups.

### Strength and limitations

By matching health claims with clinical data we were able to study cost consequences of treatment in a real life setting in a straightforward fashion. This dataset allows studying and contextualising the variability of costs as seen in the health claims with help of the clinical information that is available. The findings of this study are also useful to improve the understanding of healthcare delivery in the whole country and help improving the interpretation of health claim data of the health insurer. By identifying patients who had switched treatment prior the study entry or during the observation period, we were able to assess three different treatment groups: the non-switched ranibizumab and aflibercept groups and the (heterogeneous) group of treatment switchers. This excluded an important possible source of bias. However, this study also has its limitations. Data collected in the daily routine are inferior to those from clinical studies adhering to strict protocols. While missing data were uncommon, findings from repeated fundus fluorescein angiography examinations and also the rationale to switch treatment were unavailable, as they are not performed and recorded in a systematic fashion in clinical routine. Second, matching of the two datasets was problematic sometimes, because the entries were inconsistent. Although we carefully validated thousands of records by hand, we cannot fully exclude that the analysis file contained small errors. Nevertheless, we believe that this does not jeopardize our results. Although we included patients with DME and CRVO we were unable to perform meaningful statistical comparisons against AMD due to the limited number of participants in these two groups. By treating patients with a pro re nata scheme irrespective of drug they received, we may have equalized the number of intravitreal injections. Finally, as only one clinic was involved in this study, generalisations of the findings may be limited.

### Implications for research

Health service research is urgently needed to understand and improve clinical care [13]. These studies allow validating the expectations that were met when approving a new drug based on the results of clinical studies. Previous research has clearly highlighted the gap between the trial world and clinical reality of treatment delivery after the approval [14]. Second, the collaboration between all involved stakeholders should be intensified to tackle the challenges of a healthcare system. Real-world studies like ours contribute to the transparency within the healthcare system which ultimately serves to the advantage of the patients.

### Implications for practice

The clinical equality of the two treatment substances in AMD patients, supporting our previous results and also the findings of Johnston et al. [10] are the most interesting finding of this study The higher costs of aflibercept treatment, remains incompletely understood.

## Conclusions

Both currently licensed anti-VEGF medications showed similar clinical outcomes, and were equally expensive. These findings contradict previous studies and also the findings of those trials that were used to negotiate reimbursement in Switzerland. By linking health care claims to clinical data, this study succeeded to examine and interpret routine clinical care.

## Abbreviations

AMD: Age-related macular degeneration
Anti-VEGF: Anti-vascular endothelial growth factor
CHF: Swiss Francs
CRVO: Central vein occlusions
DME: Diabetic macular edema
IVI: Intravitreal Injection
OCT: Optical coherence tomography
PRN: Pro re nata
